# Time for Chocolate: Current Understanding and New Perspectives on Cacao Witches’ Broom Disease Research

**DOI:** 10.1371/journal.ppat.1005130

**Published:** 2015-10-22

**Authors:** Paulo José Pereira Lima Teixeira, Daniela Paula de Toledo Thomazella, Gonçalo Amarante Guimarães Pereira

**Affiliations:** 1 Department of Biology, University of North Carolina at Chapel Hill, Chapel Hill, North Carolina, United States of America; 2 Department of Plant & Microbial Biology, University of California, Berkeley, Berkeley, California, United States of America; 3 Laboratório de Genômica e Expressão, Departamento de Genética, Evolução e Bioagentes, Instituto de Biologia, Universidade Estadual de Campinas (UNICAMP), Campinas, São Paulo, Brazil; Duke University Medical Center, UNITED STATES

*Theobroma cacao* is a tropical understory tree that is one of the most important perennial crops in agriculture. Treasured by ancient civilizations in Mesoamerica for over 3,000 years, the cocoa bean now supports a multibillion-dollar industry that is involved in the production and commercialization of chocolate, a treat appreciated worldwide. The cacao tree is originally from the Amazon rainforest and is currently grown in more than 50 countries throughout the humid tropics, serving as a major source of income for over 40 million people. Each year, more than 3 million tons of cocoa beans are produced, mostly by smallholder farmers in areas of high biodiversity. Notably, the cacao tree does not require direct sunlight and naturally grows under the canopy of other, taller trees. This characteristic often encourages farmers to preserve existing forests and to plant additional trees to shelter their cacao plants [1], thereby reducing the environmental impacts of cacao cultivation. Despite its great importance, the cacao tree is affected by a number of untreatable diseases that reduce fruit production and threaten our global supply of cacao. Among them, witches’ broom disease (WBD) stands out as one of the most severe problems that affect this crop, accounting for production losses of up to 90%.

## WBD Is a Devastating Tropical Disease

WBD was first described in 1785 by the naturalist Alexandre Rodrigues Ferreira during his epic expedition across the Amazon basin. This disease is currently present in nearly all cacao-producing countries in the Americas, and it considerably reduces fruit production and bean quality, making it a major limiting factor for cacao cultivation. A striking example of the devastating impact of WBD occurred in Brazil [2], which was one of the largest cocoa exporters by the end of the 1980s. The introduction of the disease in its main producing area (the state of Bahia) in 1989 decreased the production by 70%, turning the country into a net importer of cocoa beans. Consequently, many farms were abandoned and workers were forced to move to cities that were not prepared to receive such a migration, resulting in a scenario of intense poverty and misery that persists today. Moreover, much of the Atlantic rainforest that protected cacao farms was replaced by pasture. Almost three decades after the emergence of WBD in Bahia, the Brazilian cocoa production has not yet recovered from the significant negative impact of the disease, reaching in 2013 only 65% of what was produced in 1989. Fortunately, WBD is still absent from West Africa, which currently accounts for nearly 70% of all cacao produced in the world. However, the introduction of this disease in African countries poses a real threat that would decimate the worldwide chocolate industry and cause severe socioeconomic damage. This is especially relevant because most of the cacao cultivated in Africa belongs to the highly susceptible “Amelonado” genetic group.

## WBD Is Caused by a Peculiar Fungal Pathogen

**Fig 1:**
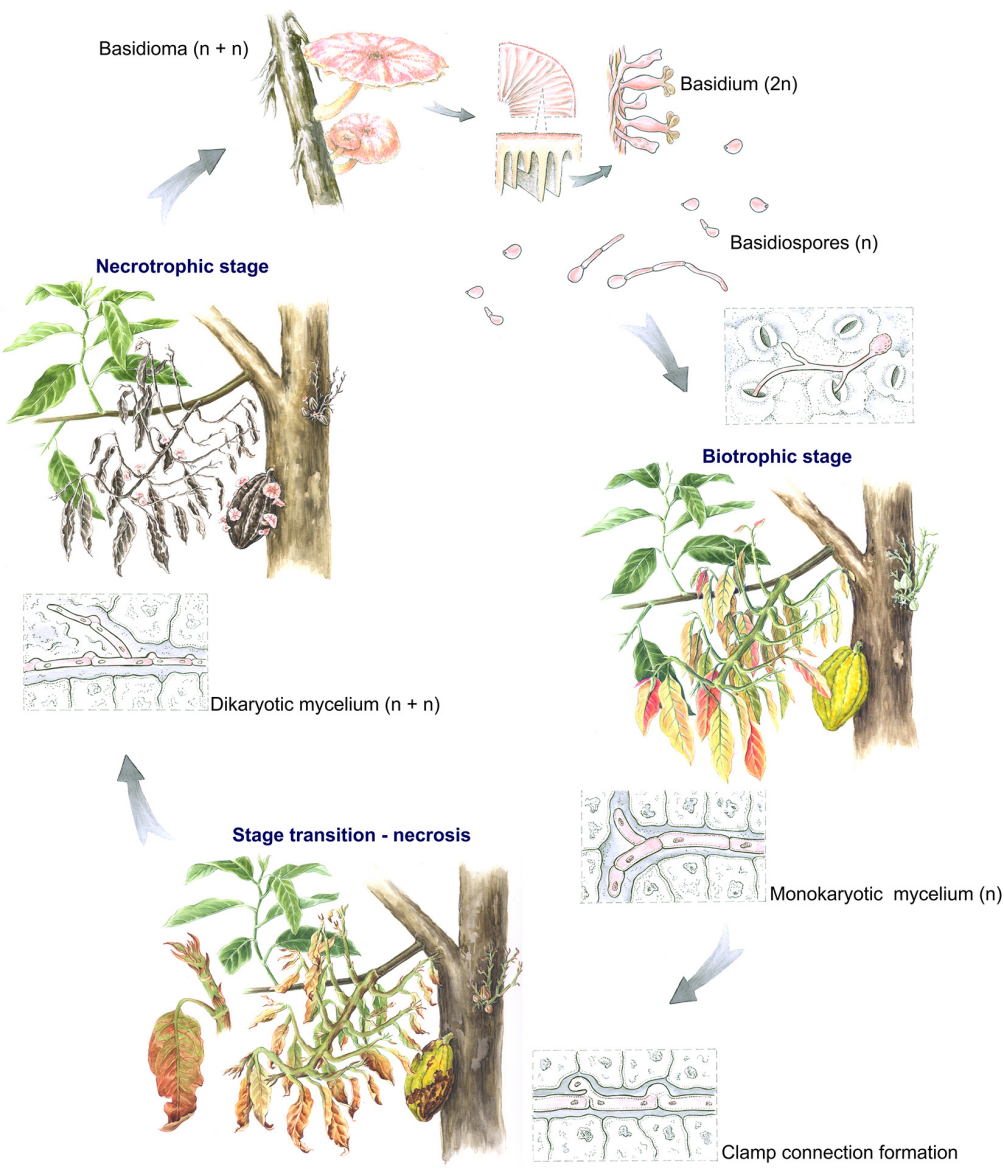
The *Moniliophthora perniciosa* life cycle in *Theobroma cacao*. Infection begins when fungal basidiospores penetrate the plant through stomata or wounds. In the first stage of the disease, *M*. *perniciosa* develops as a swollen monokaryotic mycelium that grows exclusively in the extracellular space of the living plant tissue. Infection of shoots induces drastic morphological alterations resulting in the characteristic “green broom” structure, though infection can also occur in other tissues (fruits and flowers). After one to three months of biotrophic infection, necrosis of the plant tissue occurs, giving rise to the “dry broom” structure. Necrotic tissue is colonized intracellularly by thin dikaryotic mycelium, which is characterized by the presence of clamp connections—a cross structure formed by hyphal cells that ensures the presence of two nuclei in each fungal cell. After alternating rainy and dry periods, basidiomata are formed from necrotrophic hyphae, completing the pathogen life cycle. Illustrations by Diana Carneiro.

Witches’ broom disease is caused by the basidiomycete *Moniliophthora perniciosa*. This pathogen displays a hemibiotrophic lifestyle, meaning that it initially grows on the living cacao tissues (biotrophic stage) before killing and feeding off of the dead tissue (necrotrophic stage) [3]. In comparison to other hemibiotrophic interactions, the *M*. *perniciosa* life cycle is regarded as atypical. Whereas most hemibiotrophs display a short and asymptomatic biotrophic stage (lasting for only a few days), *M*. *perniciosa* establishes a long-term biotrophic interaction with cacao that lasts for one to three months and is responsible for the main symptoms of the disease. These symptoms include hyperplasia and hypertrophy of infected tissues, loss of apical dominance, and proliferation of axillary shoots, resulting in the formation of abnormal stems called green brooms. Eventually, the green broom becomes necrotic and dies, and after alternate rainy and dry periods, dry brooms produce basidiomata, completing the fungal life cycle (Fig 1). Notably, infection of the shoots’ apical and axillary meristems results in the most distinctive symptoms of WBD. However, *M*. *perniciosa* can also infect other parts of the plant, including developing flowers and fruits.

To invade the host plant, *M*. *perniciosa* does not form specialized infection structures (e.g., appressorium), but it penetrates cacao tissues through wounds and stomata. Germinated basidiospores form swollen monokaryotic hyphae that grow intercellularly and feed on nutrients derived from the plant apoplast. In contrast with many (hemi)biotrophs, *M*. *perniciosa* does not employ nutrient-absorbing structures (e.g., haustorium and invasive hyphae), and it manipulates host metabolism to increase nutrient availability in the site of infection. In the late stages of WBD, *M*. *perniciosa* grows intracellularly as a necrotrophic mycelium, which is dikaryotic and exhibits clamp connections for nuclear transfer (Fig 1) [4].

**Fig 2:**
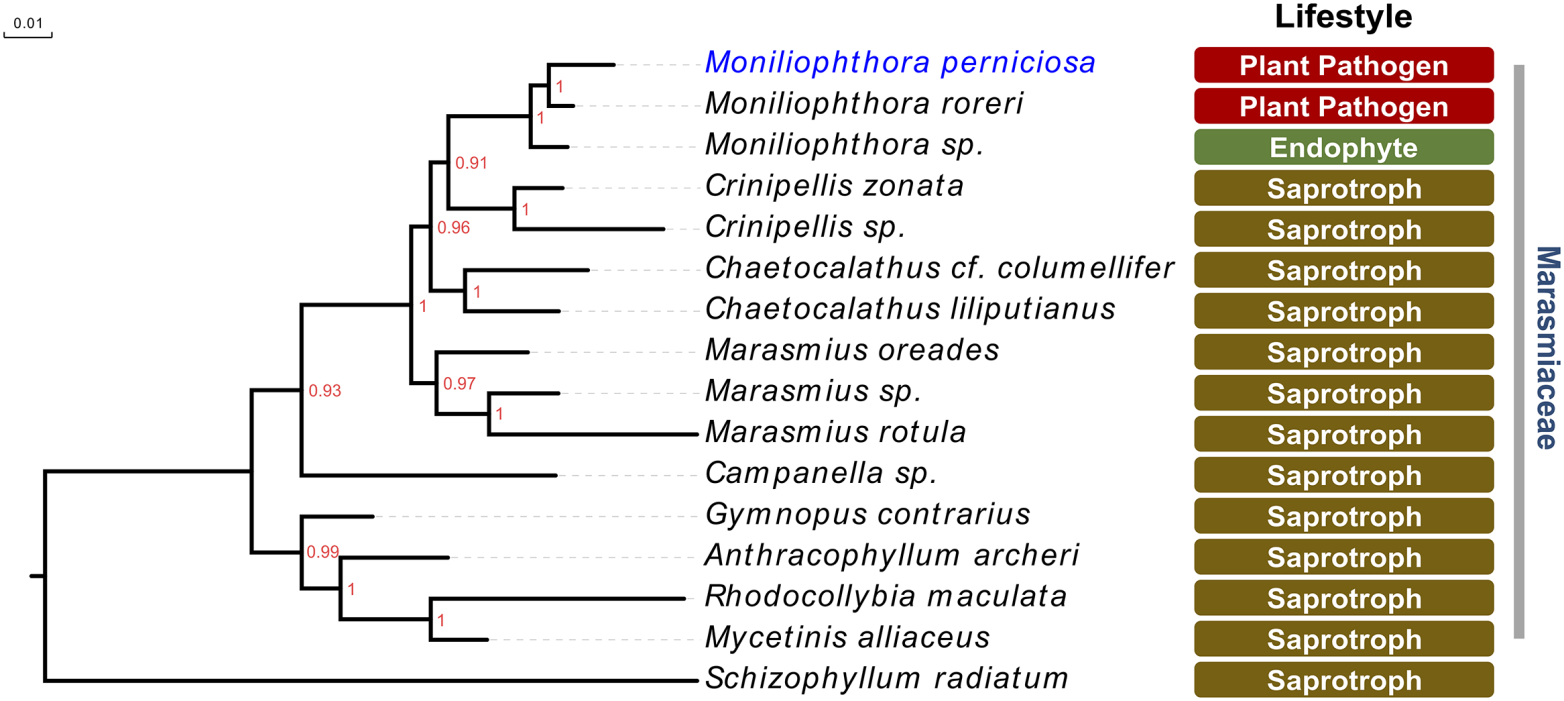
The pathogenic lifestyle of *M*. *perniciosa* is an exception within the Marasmiaceae family of basidiomycetes, which is mostly composed of saprotrophic litter and wood-decomposing fungi. The genus *Moniliophthora* includes the hemibiotrophic sister species *M*. *perniciosa* and *M*. *roreri*, the two major pathogens of *Theobroma cacao*. Notably, it also encompasses a still poorly characterized grass endophyte, suggesting that the pathogenic lifestyle of *M*. *perniciosa* may have evolved from an endophyte ancestral. The tree was constructed based on Bayesian inference using regions of the genes 25S, 18S, ITS/5.8S and Rbp1 (large fragment of the RNA polymerase II). Sequences were retrieved from Aime & Phillips-Mora (2005) [5] and Matheny et al. (2006) [34]. Numbers next to the branches represent the posterior probabilities. The species *Schizophyllum radiatum* was used as outgroup.

In addition to its intriguing life cycle, there are some other remarkable features of *M*. *perniciosa* biology and pathogenicity. Although displaying a pathogenic lifestyle, *M*. *perniciosa* belongs to the family Marasmiaceae, which is known for its predominantly saprotrophic species (Fig 2). Interestingly, the closest species to *M*. *perniciosa* evolutionarily, *M*. *roreri*, is also a cacao pathogen that exclusively infects the host fruits. The genus *Moniliophthora* also includes a grass endophyte that was isolated in New Mexico, suggesting that the pathogenic lifestyle in this group may have evolved from a biotrophic ancestor [5]. More recently, a saprotrophic *Moniliophthora* species, named *M*. *canescens*, was isolated in Asia [6]. Additional *Moniliophthora* species need to be identified and characterized in order to fully support hypotheses regarding the evolution of pathogenicity in this genus. Even so, *M*. *perniciosa* and its related Marasmiaceae species constitute a very interesting model by which to understand the evolution of pathogenicity in fungi.

Also intriguing is the fact that, in addition to cacao, *M*. *perniciosa* is found in association with other plant species in the genus *Theobroma* and in plants from unrelated families, such as Solanaceae. Based on that, *M*. *perniciosa* is classified in three biotypes according to host specificity: the C-biotype infects plants of the family Malvaceae (e.g., cacao), the S-biotype infects members of the family Solanaceae (e.g., tomato), and the L-biotype is found in association with members of the family Bignoniaceae (e.g., lianas) [7–9]. Remarkably, there is no evidence that these biotypes constitute separate species. In particular, biotypes C and S seem to be very close genetically, and they even cause similar symptoms in the host plant, indicating that they might employ conserved pathogenicity strategies. Despite the evident scientific relevance, the mechanisms associated with host adaptation are still poorly understood and thus present an exciting topic for future research.

## Important Advances in WBD Research

WBD has been the focus of attention from the scientific community for several decades. Early studies performed mostly in the 1980s and 1990s were of great importance to establishing basic knowledge about the biology of *M*. *perniciosa*, including the characterization of its hemibiotrophic lifestyle and the infection process in cacao [3,4,10–12]. Molecular biology research on this pathogen started mainly with the WBD Genome Project, an initiative launched by Brazilian laboratories in 2000 that unveiled the first DNA sequences of *M*. *perniciosa* [13]. This initiative revealed a number of fungal genes with potential roles in WBD, which have been the focus of several specific studies [14–18]. Likewise, the cacao genome sequence was released in 2010 and has been a valuable resource for WBD research [19,20].

Until the mid-2000s, our knowledge regarding the interaction between *T*. *cacao* and *M*. *perniciosa* was quite incipient. In 2005, a comprehensive biochemical characterization of WBD progression provided a first big picture of this complex pathosystem [21]. Subsequent studies also increased our knowledge about various aspects of the disease, including the importance of plant sugars as signaling molecules in WBD, mechanisms regulating the phase transition process in *M*. *perniciosa*, and the role of some fungal proteins in *M*. *perniciosa* virulence [16,22,23]. In particular, NEPs (Necrosis and Ethylene-inducing Proteins) constitute a very intriguing class of proteins, since they induce necrosis in plant tissues and seem to have a major role in the death of cacao tissues during the development of WBD [18,24]. Remarkably, Tiburcio et al. (2010) verified that *NEP* genes in *M*. *perniciosa* (and in its sister species *M*. *roreri*) were acquired by horizontal transfer from oomycetes of the genus *Phytophthora* [25]. Indeed, several *Phytophthora* species infect cacao, supporting the idea that these oomycetes coexisted with an ancestral *Moniliophthora* species. It seems likely that acquisition of *NEP* genes was an important event in the development/improvement of a pathogenic hemibiotrophic lifestyle in the genus *Moniliophthora*.

Another fungal gene characterized during the WBD research program was the *MpAOX* gene, which encodes a mitochondrial alternative oxidase (AOX) [16]. AOX constitutes an alternative respiratory route that is resistant to many inhibitors of the main respiratory chain, such as the plant defense molecule nitric oxide and strobilurin fungicides. Remarkably, the biotrophic and necrotrophic stages of *M*. *perniciosa* seem to employ different respiratory pathways: whereas the monokaryotic (biotrophic) hyphae use the alternative route, the dikaryotic (necrotrophic) hyphae rely mostly on the main respiratory chain. Notably, pharmacological inhibition of AOX completely blocks the development of the monokaryotic mycelium, whereas the dikaryotic stage is still able to grow in the presence of AOX inhibitors. Moreover, inhibition of the main respiratory pathway in monokaryotic hyphae delays the transition to the dikaryotic stage, indicating that the phase transition in *M*. *perniciosa* is linked to the cell energetic status. Importantly, the dual inhibition of the main and alternative respiratory chains blocks fungal development and represents a promising alternative to control WBD [16].

Recently, a dual RNA-seq analysis of the biotrophic stage of WBD allowed an unprecedented characterization of the transcriptomes of both the cacao plant and *M*. *perniciosa* during their interaction [26]. Significant transcriptional alterations that correlate with symptom development were identified in infected plants, as well as a set of putative fungal pathogenicity factors. The transcriptional reprogramming associated with hormonal metabolism was remarkable and is compatible with auxin, gibberellin, cytokinin, and ethylene unbalance. Interestingly, auxin-responsive genes were strongly up-regulated in green brooms, but plant genes required for the biosynthesis of this hormone were not differentially expressed. These data indicate that *M*. *perniciosa* may interfere directly with the cacao hormonal metabolism, which is in agreement with the finding that this fungus is able to produce the plant hormone auxin [27]. In addition, a clear carbon deprivation signature in the transcriptomes of infected plants was verified, including the up-regulation of the glyoxylate cycle, lipid degradation, asparagine biosynthesis, and reduction of photosynthetic rates. This nutrient-starving condition appears to trigger a premature senescence process in infected plant tissues, which is responsible for the first signs of necrosis observed during WBD. Based on this transcriptomic analysis, we now have a model of the molecular events underlying the biotrophic stage of WBD as well as new insights on the plant metabolic processes associated with the transition to the dry broom/necrotrophic stage of the disease.

This detailed transcriptional analysis of the biotrophic stage of WBD is part of the WBD Transcriptome Atlas initiative (http://www.lge.ibi.unicamp.br/wbdatlas). This database comprises a continuously growing set of RNA-seq libraries covering the transcriptomes of both *M*. *perniciosa* and *T*. *cacao* across multiple biological conditions (e.g., pathogen life cycle and stages of WBD). With this initiative, we aim to provide the WBD research community with access to a valuable resource that might be used as an additional line of evidence in the study of gene function.

## Searching for WBD-Resistant Varieties

The development of resistant cacao varieties can be a durable and sustainable approach to meet the increasing demand for cocoa beans. The genetic improvement of disease resistance in cacao started in the 1930s, when Frederick J. Pound undertook expeditions to the Upper Amazon region to search for wild cacao trees with an absence of WBD symptoms. Since then, many of these varieties have been used in breeding programs for WBD resistance. Notably, Scavina 6 (SCA6) has been one of the most widely used sources of resistance to WBD identified so far. However, because of its poor agronomic characteristics, it is not directly used as a clone, but is usually crossed with other high-quality varieties in order to obtain hybrids with disease resistance as well as high yield and large bean weight.

Although SCA6 has been a recognized source of WBD resistance, it is susceptible to *M*. *perniciosa* in some regions of Ecuador, Peru, and Brazil. This geographical variation in disease susceptibility is assumed to be due to regional variability in the pathogen populations. In this regard, the exploration of novel cacao varieties is essential to expand the genetic resources of WBD resistance. Indeed, high resistance to WBD has been identified in other germplasm groups. A study by Sereno et al. (2006) identified resistant accessions in wild germplasm originally collected from distinct river basins in the Brazilian Amazon [28]. These resistant accessions are CAB0208 and CAB0214 and constitute promising parental lines to be used in breeding programs. Another outstanding source of resistance to WBD is the CCN51 variety, which is the product of an independent breeding program developed in the 1960s by Homero Castro in Ecuador. Despite its poor organoleptic quality, CCN51 is valued for its high productivity and disease resistance, which make it another promising parental line to be used in the cacao-breeding programs worldwide. So far, the molecular basis of WBD resistance in all these varieties remains completely unknown, and a classic gene-for-gene relationship between plant resistance and fungal avirulence genes remains to be identified and characterized.

## Bottlenecks in WBD Research and Exciting New Directions

WBD results from the interaction between two non-model organisms. Therefore, a number of methodological tools that are commonly used for model organisms are still missing in this system. In particular, the routine application of genetic manipulation techniques constitutes the main bottleneck in WBD research. In 2009, RNAi-based gene silencing was reported for the necrotrophic mycelium of *M*. *perniciosa* [29]. However, stable gene silencing could not be obtained in the infective stages of the pathogen. More recently, Barau et al. transformed *M*. *perniciosa* protoplasts with a cassette containing the hygromycin resistance gene and a Green Fluorescent Protein—tagged version of the *ATG8* gene [23]. Even so, genetic manipulation is not a standard practice in *M*. *perniciosa*, and a number of basic methods still need to be established and validated. Particularly, gene-specific mutagenesis has never been reported, which limits the study of gene function in this pathogen. The development of the CRISPR/Cas9 editing tool provides new perspectives and stands as a very promising approach for efficient genetic manipulation of *M*. *perniciosa*.

Transformation protocols have already been developed for cacao somatic embryos [30]. Nevertheless, as a perennial plant, the cacao tree has a quite long life cycle (3–4 years from seed to seed), which considerably hinders the use of classical genetics approaches in the study of this plant. In this context, the infection of tomatoes by isolates belonging to the S-biotype of *M*. *perniciosa* presents a more amenable and promising model system for WBD [31,32]. In this regard, it is of primary importance to understand how the disease development compares in tomatoes and cacao. Comparative genomics and transcriptomics of *M*. *perniciosa* isolates belonging to different biotypes offers an exciting direction in which to expand our knowledge of the mechanisms involved in host adaptation in this pathogen.

Finally, results from the WBD research program may eventually translate into strategies to control the disease more efficiently. Several strategies have been developed and tested with limited success over the past years. Currently, two promising approaches include the following: (1) the use of biofungicides based on the mycoparasite *Trichoderma stromaticum*, which can antagonize *M*. *perniciosa* [33], and (2) the use of drugs to simultaneously block the main and the alternative respiratory chains of the pathogen, which showed high effectiveness in in vitro assays [16]. These strategies must be carefully evaluated under field conditions and, along with the disciplined management of the farms, they may constitute important approaches to fight off WBD in the near future. Moreover, the characterization of resistance genes (e.g., Nucleotide-Binding Leucine-Rich Repeats [NLRs]) in resistant cacao cultivars (or even in its related wild plant species) may, through biotechnology, provide additional effective ways to tackle WBD in the long term. With the advent of the CRISPR/Cas9 technology, the development of disease-resistant cacao plants is certainly a very promising and sustainable strategy to control WBD. However, the success of this strategy still depends on the regulation of this new technology and on the public’s approval.
